# Breaking down the relationship between disruption scores and citation counts

**DOI:** 10.1371/journal.pone.0313268

**Published:** 2024-12-19

**Authors:** Mingtang Li, Giacomo Livan, Simone Righi

**Affiliations:** 1 Department of Computer Science, University College London, London, United Kingdom; 2 Dipartimento di Fisica, Università degli Studi di Pavia, Pavia, Italy; 3 Department of Economics “Marco Biagi”, University of Modena and Reggio Emilia, Modena, Italy; Leiden University: Universiteit Leiden, NETHERLANDS, KINGDOM OF THE

## Abstract

The emergence of the disruption score provides a new perspective that differs from traditional metrics of citations and novelty in research evaluation. Motivated by current studies on the differences among these metrics, we examine the relationship between disruption scores and citation counts. Intuitively, one would expect disruptive scientific work to be rewarded by high volumes of citations and, symmetrically, impactful work to also be disruptive. A number of recent studies have instead shown that such intuition is often at odds with reality. In this paper, we break down the relationship between impact and disruption with a detailed correlation analysis in two large data sets of publications in Computer Science and Physics. We find that highly disruptive papers tend to receive a higher number of citations than average. Contrastingly, the opposite is not true, as we do not find highly cited papers to be particularly disruptive. Notably, these results qualitatively hold even within individual scientific careers, as we find that—on average—an author’s most disruptive work tends to be well cited, whereas their most cited work does not tend to be disruptive. We discuss the implications of our findings in the context of academic evaluation systems, and show how they can contribute to reconcile seemingly contradictory results in the literature.

## Introduction

In an increasingly competitive academic environment, the quality of scientific output and the performance of researchers are constantly monitored, quantified, and ranked across a variety of aspects. Some of these can be measured rather objectively (e.g., productivity, citation counts, ability to attract funding, etc. [1–3]), while others are more elusive, such as the ability to disrupt and/or to produce impactful research [4, 5]. Conventionally, the most prevalent metrics in bibliometric analyses are citations and citation-based indicators [6–8]. These include simple citation counts as well as more sophisticated bibliometric indicators, such as the well-known *h*-index [9], *g*-index [10], or indicators of an author’s performance relative to their field (see, e.g., [11]). These metrics reflect the extent to which research outputs are recognized by the scientific community. They also play an increasingly pervasive role in research evaluation systems, influencing research rankings, grant allocations, tenure and promotion decisions [12–16].

However, as the reliance on citations has grown, so too has the scrutiny regarding their use and implications. Nowadays, citation-based metrics have been increasingly scrutinized by the academic community and have become somewhat controversial [12, 17–20]. One of the major concerns is that such indicators—and citations in general—primarily measure the amount of academic impact [12, 17, 21]. However, academic impact is a multifaceted concept encompassing various dimensions of scientific publications [22–25]. In particular, citations fall short in capturing the nature of academic impact [21], which is more accurately reflected by an emerging indicator for scientific disruption, known as the CD index [26] or the disruption score [27].

The fundamental idea of the disruption score is that a highly disruptive paper can eclipse attention towards its prior works, making subsequent publications more inclined to cite the focal paper rather than the references listed in its bibliography [26]. Since its introduction by Funk *et al*. [26], the disruption score has been employed in a variety of studies [27–30], demonstrating its effectiveness to distinguish between disruptive and developmental contributions [27–30]. Following its surging popularity, a number of variants has been developed to adjust certain aspects of the original metric [26, 31, 32]. For example, Wu *et al.* explore several indicators of disruption by manipulating the one from [26] and suggest removing the citation impact of prior works as calculated from the bipartite network of the focal paper [32]. A recent study compares these variants with peer review assessments and shows that they essentially measure similar latent information in medical data, with none proving significantly superior to the others [33]. Based on these observations, we will focus on the original version of the disruption score, as its robustness has been validated in a variety of empirical studies [26, 27, 29].

Prior to the introduction of the disruption score, early efforts to quantify scientific breakthroughs proposed novelty metrics in bibliometric studies, which conceptualize new discoveries in science as a recombination of existing pieces of knowledge [34–39]. Within this framework, Uzzi *et al.* assess novelty by analyzing references cited by a paper, focusing on how typical or atypical the combinations of these references are (as represented by journal pairs) [38]. Similarly, Wang *et al.* use journal pairs to study knowledge recombination, but they place more emphasis on previously unseen journal pairs in bibliographies [39, 40]. The relationship between novelty and citation has also been examined in various studies. These studies identify an inverted U-shaped relationship between these metrics [38, 41], suggesting that the most-cited papers often draw heavily from conventional combinations of research while also incorporating unusual knowledge combinations [38, 42]. Other studies use regression analysis to investigate this relationship, finding that papers featuring novel or rare knowledge combinations are more likely to receive a higher number of citations [39, 43, 44].

Although both novelty metrics and the disruption score are designed as tools to evaluate the innovativeness of scientific output, research has shown that they essentially capture different types of information. Theoretically, novel contributions integrate previously unconnected knowledge areas, focusing on the recombination of existing elements [38]. By comparison, disruptive research disrupts prevailing theories and introduces new ideas, emphasizing paradigm shifts and breaking existing norms [26]. This implies a fundamental difference between the two metrics [45, 46]. In terms of empirical evidence, Leahey *et al.* identify three types of novelty: new results, new theories, and new methods. They explore which types are more disruptive, discovering that new methods are typically more disruptive, new theories less so, and new results do not have a robust effect [21]. These insights, along with other empirical findings, suggest that novelty indicators and the disruption score should be considered as different metrics [21, 41, 45].

Treating the disruption score as different from the novelty metrics suggests that the aforementioned novelty-citation relationship does not apply to the disruption score. Meanwhile, the increasing number of studies on the disruption score underscores the importance of exploring its relationship with citation counts [27, 29, 30, 47, 48]. However, current research lacks a systematic analysis of this relationship and some studies present conflicting results [30, 47]. A recent investigation by Zeng *et al.* reports a negative relationship between disruption scores and citation counts [47], indicating that disruptive papers in science are losing impact over time. Conversely, Wei *et al.* analyze Nobel Prize-winning papers and find that such papers not only garner more citations but also achieve higher disruption scores [30]. These seemingly contradictory findings prompt us to examine a comprehensive relationship between disruption scores and citation counts. Additionally, several studies in science of science have employed the disruption score to examine the performance of researchers. Such studies typically involve the analysis of both the disruption score and citation counts in scientific careers [48, 49]. To pave the way for future research in career analysis, we also seek to expand the disruption-citation relationship to the careers of researchers.

In this paper, we explore a comprehensive relationship between disruptions and citations, as measured by disruption scores and citation counts. Our analysis is conducted at two levels: papers and careers. To study this relationship, we propose two essential research questions: (1) Are disruptive papers highly cited?; and (2) Are highly cited papers disruptive? We address the first question by breaking down the correlation between disruption scores and citation counts across each cumulative percentile of the top disruptive papers. Then, we answer the second question by investigating whether the most cited papers are disruptive. Finally, we extend our paper-level analysis to careers, examining the relationship between disruption scores and citation counts within the publication sequences of long-lived researchers.

## Results

We collect papers published between 1986 and 2015 in Computer Science and Physics, from the AMiner citation network dataset (version 12) and the Web of Science database, respectively (see Materials and methods). We quantify disruptions by associating a disruption score with each paper in our datasets, and measure citations by analyzing the citation counts each paper accumulates over the first five years after publication, which is a customary proxy in the literature [29, 47, 50]. Overall, our analysis comprises 898,624 papers in Computer Science and 1,236,016 papers in Physics.

### A detailed breakdown on the correlations between disruptions and citations

We begin our analysis with a detailed breakdown of the correlation between disruptions and citations. Namely, we rank all the papers in our datasets based on their disruption scores and citation counts. In the following, we shall refer to the rankings computed via disruption scores and citations as the ‘disruption rank’ and the ‘citation rank’, respectively.

To break down the correlations, We initially select papers ranked in the top 1% within the disruption rank and identify their corresponding positions in the citation rank. The Spearman correlation coefficient for the top 1% of disruptive papers is calculated using these two position vectors. Then, we expand our analysis to include the next percentile of disruptive papers, i.e., the top 2% of disruptive papers, computing the correlation coefficient for the cumulative top 2% (including both the top 1% and top 2%) of disruptive papers. We further repeat this process for papers in the cumulative top 3%, top 4%, etc., until all papers in Computer Science (898,624 papers in total, 8,986 papers in each percentile) and Physics (1,236,016 papers in total, 12,360 papers in each percentile) have been included. Following this procedure, we analyze correlations not only for a selected subset of highly disruptive papers but also across the entire publication dataset.

**Fig 1:**
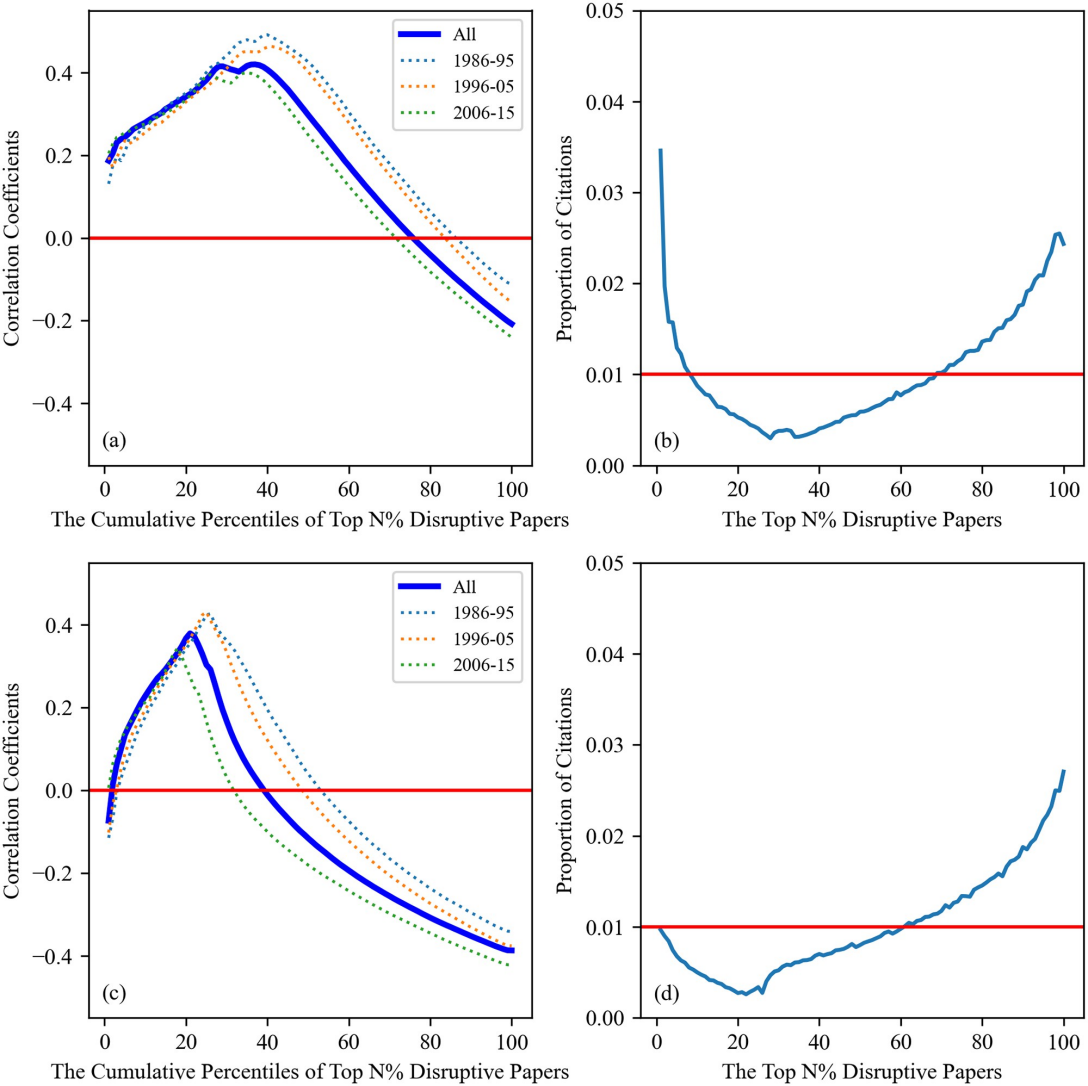
Correlation coefficients across various cumulative percentiles of disruptive papers in both disciplines. (a) Trajectory of correlation coefficients in Computer Science. The thick blue curve is derived from all publications, while dashed lines represent the correlation coefficients corresponding to the 1986–1995, 1996–2005, and 2006–2015 groups. (b) The proportion of citations received by each percentile of Computer Science papers. The correlation pattern observed in (a) can be explained by the proportion of citations received as shown in (b). (c) and (d) are the equivalent versions of the correlation trajectory and the proportion of received citations in Physics. They can be interpreted in a similar way to (a) and (b).

In Fig 1(a)–1(c) we plot the aforementioned correlation coefficients across various cumulative percentiles of top disruptive papers in Computer Science and Physics. We observe a positive correlation coefficient between disruptions and citations for papers in the upper percentiles of the disruption rank. Such correlation increases as we incorporate more percentiles into our analysis, reaching a peak value around the top 25th percentile, then declining to negative values. To explain such a pattern, in Fig 1(b)–1(d) we report the proportion of citations received by papers in each percentile of the disruption rank. We find that the papers receiving the lowest share of citations are those around the 25th percentile, i.e., where we observe the peak in correlation between disruptions and citations. After that, less disruptive papers progressively become more cited, which causes the correlation coefficient to decrease. Eventually, the correlation coefficient becomes negative when we consider a large enough portion of papers in our dataset, which supports the result by Zeng *et al.* on the negative correlation between disruptions and citations.

Fig 1(b)–1(d) also show that the most disruptive papers are quite well recognized, as evidenced by the relatively higher proportion of citations received by the most disruptive papers in both Computer Science and Physics, although with remarkable differences. In fact, highly disruptive papers in Computer Science are cited much more frequently than one would expect from a random baseline (i.e., all percentiles receiving a 1% share of all citations). The same cannot be said for Physics, where the most disruptive papers receive citations slightly lower than the random baseline. These differences are responsible for the positive (negative) correlation between disruptions and citations observed in the top percentiles of the disruption distribution in Computer Science (Physics).

**Fig 2:**
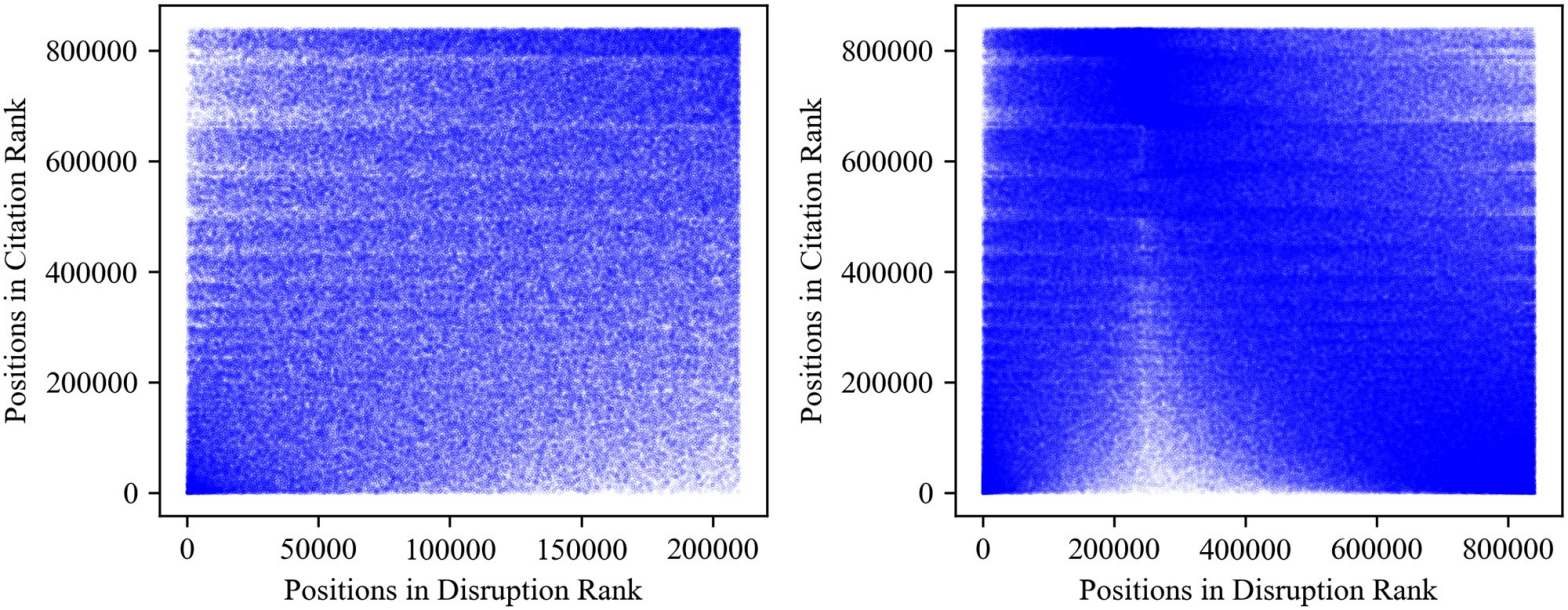
Scatter plots of the cumulative top 25% and the full dataset of disruptive papers in Computer Science. Left panel: Scatter plot of the cumulative top 25% of disruptive papers. The corresponding Spearman correlation coefficient is 0.297. Right panel: Scatter plot of the full dataset (top 100%) of disruptive papers. The correlation coefficient is -0.264.

In Fig 2, we present scatter plots depicting the cumulative top 25% (left) and the full dataset (right) of disruptive papers in Computer Science. This figure serves as an example to illustrate how the correlation trajectories are derived. In the left panel, data points primarily cluster in the lower-left and upper-right corners, suggesting a positive correlation between disruptions and citations. For these papers, the Spearman correlation coefficient is 0.297. The scatter plot on the right illustrates an ‘increase followed by a decrease’ pattern, with an overall correlation coefficient of -0.264. All these observations substantiate the correlation trajectory observed in Fig 1.

We test the robustness of the aforementioned results in the following ways. First, we split all papers in our dataset into three groups based on their publication year, namely 1986–1995, 1996–2005, and 2006–2015, and then plot the correlation trajectory for each group. The aim of this test is to illustrate that our results are robust across different time periods. As can be seen in Fig 1(a) and 1(c), we find consistent patterns across the three groups. Second, we replicate our analysis using the CD_5_ metric (see S1 Fig). This metric is computed only from papers published in the 5 years following the focal paper, which is frequently used in the literature [27, 29]. Third, we standardize the disruption score (see Materials and methods) of each paper to account for the fact that papers tend to become less disruptive over time [29]. We repeat our analysis with the standardized disruption scores, obtaining consistent results across the two disciplines (see S2 Fig). Finally, we run our analysis against a null model created by reshuffling the 5 years of accumulated citations received by each paper while keeping their disruptions intact. By reshuffling citations, we randomize the position of top disruptive papers in the citation rank, thus the new correlation coefficients are calculated under the null model. We find that the correlation patterns cannot be explained by the null model, and the correlation coefficients across different percentiles of disruptive papers are around zero (see S3 Fig).

### Most-cited papers are relatively less disruptive

After examining the relationship between disruptions and citations across various cumulative percentiles of top disruptive papers, we now explore the relationship from the perspective of citations, i.e, by analyzing different cumulative percentiles of the citation distribution. Similar to the procedure described in the previous section, we choose the top 1% most cited papers in the citation rank and identify their respective positions in the disruption rank. We calculate the correlation coefficient between these two position vectors and repeat the procedure for the cumulative top 2%, 3%, up to all the papers in both Computer Science and Physics.

**Fig 3:**
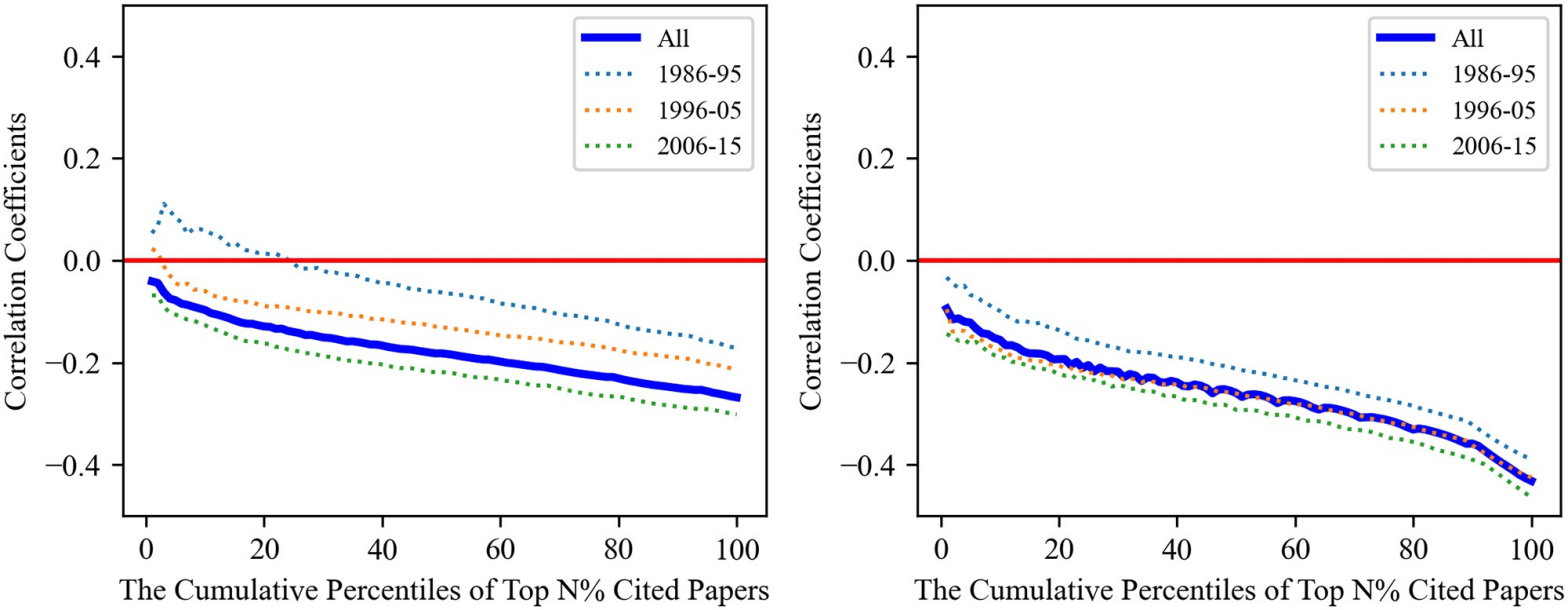
Correlation coefficients across various cumulative percentiles of the most-cited papers. In both Computer Science (left) and Physics (right), the patterns of correlation coefficients are very similar, which indicates that the most-cited papers tend to be less disruptive.

As shown in Fig 3, a negative correlation coefficient is apparent across cumulative percentiles of the highly cited papers in both disciplines. The negative correlation strengthens as we incorporate more percentiles of papers into our analysis. Such a pattern indicates that the most-cited papers tend to be less disruptive. Moreover, in both disciplines, the correlation coefficients are generally higher for papers published between 1986 and 1995. In particular, we can find a positive correlation coefficient in the top 1%-30% of the most-cited Computer Science papers. By contrast, for papers published in more recent decades (1996–2005, 2006–2015), the correlation trajectory is not only negative over the entire distribution, but the negative correlation becomes even more pronounced. To further corroborate these results, we perform the same analysis with the CD_5_ metric, the standardized disruption scores, and the null model. Our results are valid under all these robustness checks (see S4 Fig).

### The relationship between disruptions and citations in scientific careers

Having established the paper-level relationship, we then extend our investigation to the relationship between disruptions and citations in scientific careers. To construct author datasets, we match each paper in our datasets with its respective authors and then identify long-lived researchers with an active publication record. Specifically, we only include in our analysis authors who started their careers between 1980 and 2000 and had an academic career of at least 20 years. Among these authors, we retain only those who published more than 10 papers, with a publication frequency of at least one paper every 5 years (in line with [51]). Based on these selection criteria, we are left with 27,598 Computer Scientists and 34,527 Physicists (see Materials and methods).

**Fig 4:**
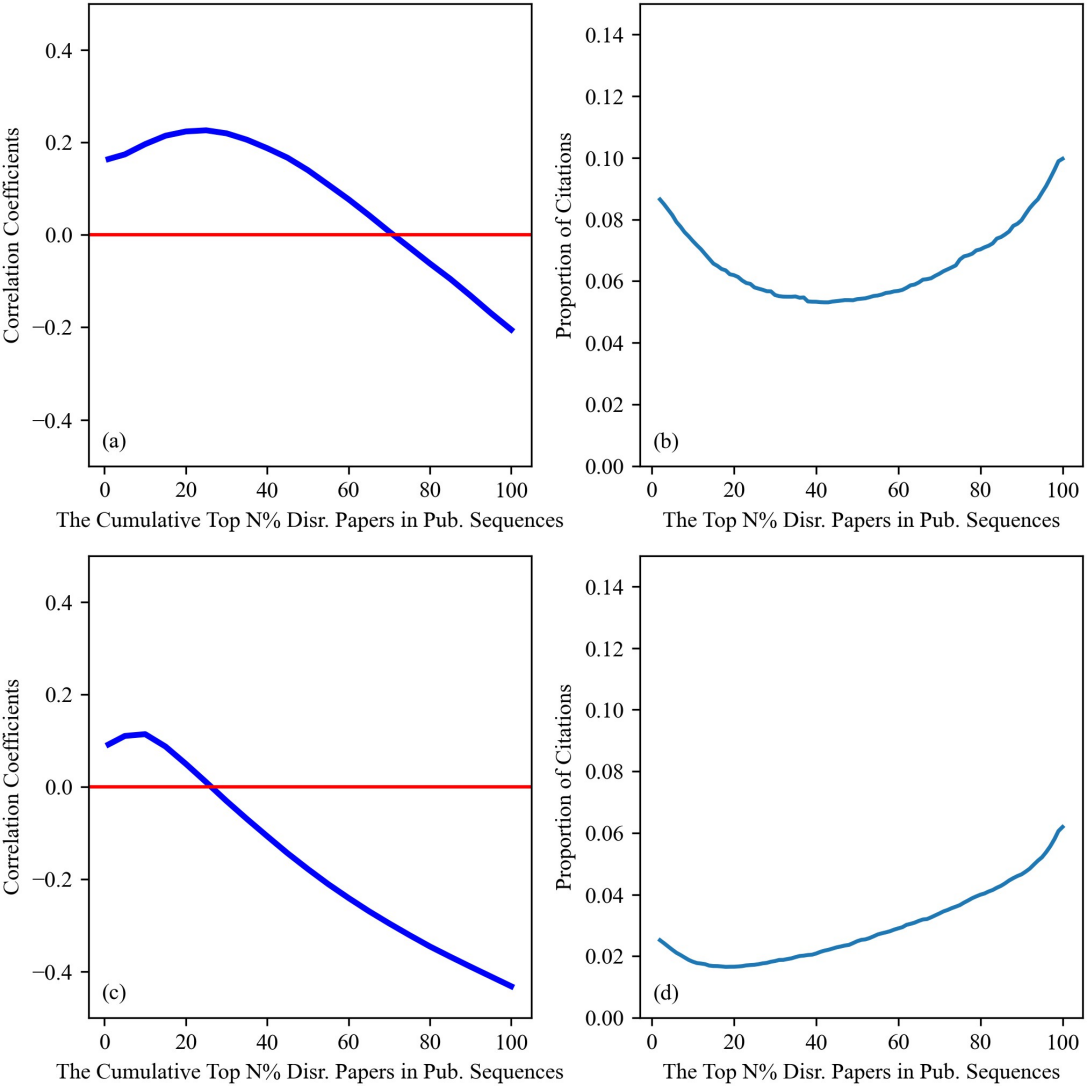
Average values of correlation coefficients across various cumulative percentiles of disruptive papers in scientific careers. (a) The average values of correlation coefficients in the careers of Computer Scientists. (b) The average values of the proportion of citations received by papers at each percentile in the publication sequence of Computer Science researchers. Again, the correlation pattern observed in (a) can be explained by the proportion of citations received as depicted in (b). (c) and (d) are the equivalent version of (a) and (b) in Physics. We can observe that in both disciplines, our paper-level results are consistent in the career-level analysis.

We first generalize the findings concerning the disruptive papers to scientific careers. In line with the method outlined in the corresponding paper-level analysis, we start by creating disruption and citation ranks for our pool of researchers. Within each researcher’s publication sequence, we locate the positions of top 1% disruptive papers within both disruption and citation ranks and compute the correlation coefficient between these two sets of positions. It should be noted that in this analysis, correlation coefficients are not computed for each cumulative percentile to avoid an excessive amount of repeated values, which arises from the relatively small number of publications (compared to the paper datasets) a researcher can publish. Instead, this process is applied selectively for the cumulative top 1%, 5%, 10%, 15%, etc. Following this procedure, each researcher obtains a list of correlation coefficients at various cumulative percentiles. Finally, we collate correlation coefficients for identical cumulative percentiles across all authors in our datasets, and plot the average values of these correlation coefficients in Fig 4(a) and 4(c).

Similar to the previous analysis, we illustrate the proportion of citations received by each percentile of disruptive papers at the career level in Fig 4(b) and 4(d). We observe that the overall trends in panels (b) and (d) are comparable to the trends in the paper-level results. It is noted that the curves in (b) and (d) achieve higher values compared to the results in the paper datasets. This happens because the same papers can fall within different percentiles when considering less prolific authors. When we restrict our scope of investigation to researchers who have more than 100 publications, i.e., no overlaps between percentiles, our results are very much similar to the paper-level results (see S5 Fig).

As can be seen in Fig 4, the findings we obtain here are fairly similar to those we observe at the paper level. Specifically, the most disruptive papers in the careers of Computer Scientists and Physicists still attract a relatively high proportion of citations. The correlation trajectories in scientific careers also display a pattern of initial increase followed by a decrease, and such a trajectory can also be explained by the proportion of citations received by each percentile of papers published in a career. Furthermore, we observe a negative correlation coefficient when considering all papers in a career, indicating that the overall negative relationship between disruptions and citations persists at the career level. The only significant difference between our findings in academic publications and scientific careers is that the correlation coefficients for the most disruptive papers are now positive in both disciplines. This suggests that the most disruptive papers within a career are well rewarded in terms of citations by their respective scientific communities.

**Fig 5:**
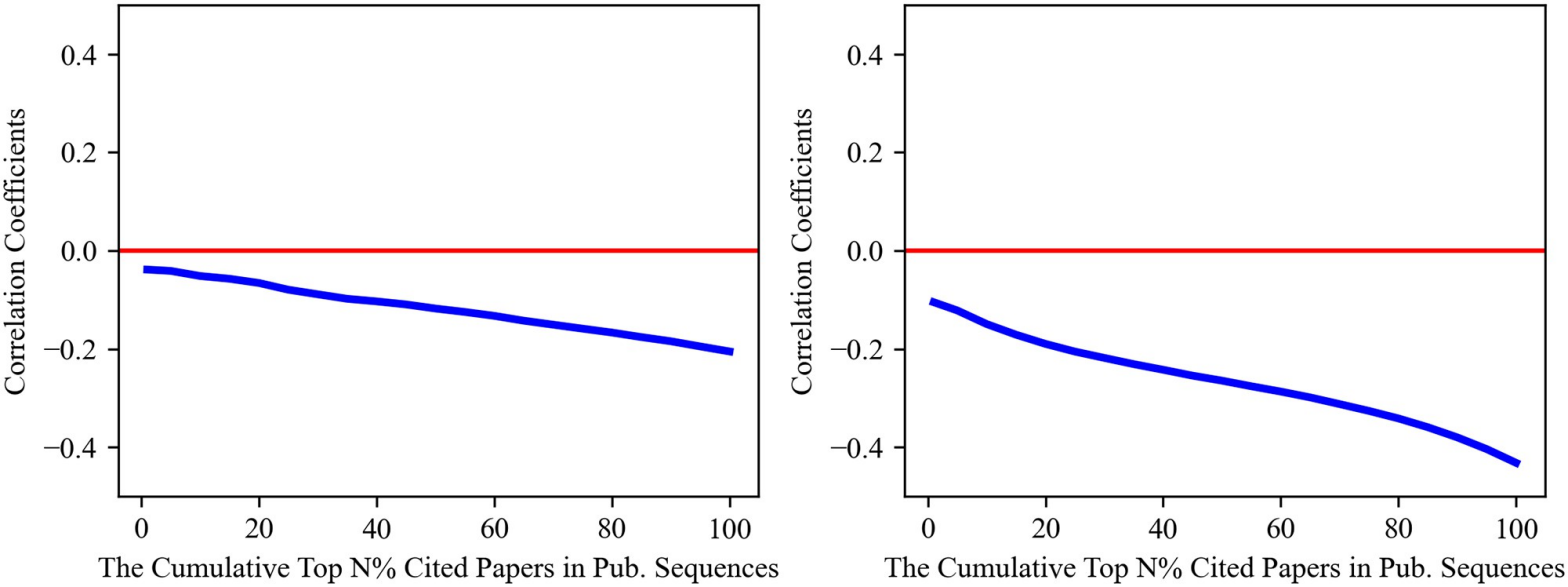
Average values of correlation coefficient across various cumulative percentiles of the most-cited papers. It can be seen that in both Computer Science (left) and Physics (right), our paper-level results hold true in scientific careers.

We then expand our results regarding the correlations for the most-cited papers to the context of scientific careers. To achieve this, we follow the steps outlined in the corresponding paper-level analysis and compute rank-rank correlations over an increasing number of percentiles of the citation distributions obtained at the career level. The results are illustrated in Fig 5. In line with our results at the paper level, we can still observe negative correlation coefficients across cumulative percentiles in the careers of both Computer Scientists and Physicists. This reinforces our conclusion that the most-cited papers tend to be less disruptive.

To substantiate our findings, we replicate our career-level study using the CD_5_ metric and the standardized disruption scores, obtaining consistent results (see S6, S7 and S9 Figs). Moreover, we construct two null models in a similar manner to the previous analysis by reshuffling the disruption score and the 5-year accumulated citations in each author’s publication sequence. We then reapply the career-level analysis utilizing these null models, and find that our conclusions cannot be explained by these null models (see S8 and S9 Figs). Based on all these results, we conclude that the paper-level relationship between disruptions and citations qualitatively holds in scientific careers.

## Discussion

This study presents the detailed relationship between disruptions and citations, measured in terms of disruption scores and citation counts, respectively. It fully captures the relationship between these two metrics through two main research questions, namely (1) are disruptive papers highly cited?; and (2) are highly cited papers disruptive?

To answer question (1), we analyze the correlation coefficients between disruption scores and citations across different cumulative percentiles of papers ranked by their disruption scores. In both Computer Science and Physics, we find that the correlation varies when we observe different samples of disruptive papers, and that the variations in the correlation coefficients can be explained by the proportion of the citations received by each percentile of papers. Our results reconcile the seemingly contradictory conclusions between Wei *et al*. [30] and Zeng *et al*. [47]. Specifically, papers with higher levels of disruption exhibit a positive correlation between disruption scores and citation counts. This pattern is consistent with the finding, e.g., that Nobel Prize-winning papers typically receive more citations and are characterized by higher disruption scores [30]. However, as we incorporate more percentiles of papers into our analysis, the correlation coefficient gradually shifts from positive to negative values, and ends up with a negative correlation when we include most of the papers in our analysis, in line with findings by Zeng *et al*. [47]. Concerning question (2), we find a negative correlation between disruption scores and citations in both disciplines, which suggests that the most-cited papers tend to be less disruptive. Moreover, we observe that such a negative correlation intensifies over time.

Having determined the relationship between disruption scores and citation counts at the level of academic publications, we then extend our analysis to the publication sequences of researchers in Computer Science and Physics, concluding that the aforementioned relationship qualitatively holds at the level of careers. Our results suggest that there are two strategies researchers might adopt to maximize their citations. The first strategy aims to publish truly disruptive papers. This strategy is beneficial to the development of science as a whole but requires researchers to accumulate research experience, go through periods of focus and low productivity [49], and undertake the risk of receiving only a limited number of citations. The second strategy is to produce papers that attract a large number of citations. Such a strategy favors the career progression of individual researchers. However, it may also incentivize researchers to focus excessively on popular research topics and incremental work, which can be detrimental to the overall diversity and innovation of scientific research [52].

A common criticism of the disruption metric is that the score of a paper can be distorted upward by receiving only a small number of citations [29], i.e., high scores do not indicate high research quality but might simply reward papers that are less appreciated by citations. However, our results show that the top 1% of disruptive papers not only achieve high disruption score levels but also attract a high proportion of citations. These findings are consistent in both Computer Science and Physics, and apply to both paper-level and career-level analysis. Therefore, such a criticism does not apply to papers with very high disruption scores.

The evaluation of research outputs is often based on bibliometric indicators of citations [53–55]. Our study reveals that papers standing out in this regime tend to be less disruptive. Similarly, if evaluations were to be purely based on disruption scores, some high-scoring papers may also exhibit limited academic influence. A more effective approach would integrate both metrics in research evaluation. Such an approach would enable us to identify papers that are both disruptive and highly cited. Papers that excel under those criteria are typically recognized as works of very high quality [30]. Therefore, we advocate that scientific evaluation should be carried out through a comprehensive analysis of publications [8, 56].

This study further substantiates the efforts to conceptualize disruption scores and citation counts as distinct dimensions of academic impact [21]. Our findings indicate that disruption scores and citation counts—the most prevalent proxy for academic impact—capture essentially different types of information. These results underscore the multifaceted nature of academic impact [22–25], providing empirical evidence to inform future scientific policies and funding decisions based on academic impact.

## Materials and methods

### Data

We collect publication and citation data pertinent for Computer Science from the AMiner citation network dataset (version 12). The AMiner dataset extracts papers published between 1960s to 2020 from DBLP, ACM, MAG, and other sources in Computer Science [57], and it records a total of 4,894,081 papers and 45,564,149 citation relationships. The AMiner dataset has been utilized in a variety of bibliometric studies [49, 58–60]. For publications in Physics, we retrieve data from the Web of Science (WOS) database. We extract the papers published by long-lived researchers who maintain an active publication record, along with the citation network related to their publications. In total, we collect 1,619,039 papers and 12,621,175 citation relationships from 1985 to 2020.

While the Computer Science AMiner dataset contains unique identifiers for each researcher, the Physics WOS database does not provide unique author identifiers. To link researchers in Physics with their respective publications, we employ the method proposed by Caron *et al.* to disambiguate author names [61]. This method determines a similarity score between pairs of authors by considering a series of attributes, such as ORCID identifiers, names, affiliations, emails, coauthors, grant numbers, etc. If a pair of authors has a higher similarity score, they are more likely to be identified as the same person. The effectiveness of this method has been validated by a recent study that employs the WOS database to compare four different unsupervised approaches to author name disambiguation. It finds that the Caron method outperforms the other methods, achieving precision and recall scores higher than 90% [62].

In our analysis, we only calculate disruption scores for papers published before 2016, thereby allowing papers in our pool to accumulate citations for at least 5 years. We set filtering criteria for researchers in line with [51], performing our career analysis on a total of 27,598 and 34,527 researchers in Computer Science and Physics, respectively.

### The disruption score

We employ the disruption score to quantify the disruption level of each paper in our datasets. The fundamental idea of the disruption score is that a highly disruptive publications can overshadow its preceding papers. The subsequent papers are more likely to cite the disruptive work over the references listed in its bibliography. The disruption score is particularly useful in differentiating between disruptive and developmental pieces of work, and it has been validated using data from academic publications, patents, and software products [26, 27, 29].

To be more specific, we create a citation network centered on a focal papers, combined with its references (preceding papers) and subsequent papers. The subsequent papers can be further categorized into three groups: papers citing only the focal paper, those citing both the focal paper and the references, and those citing only the references of the focal paper. Let us assume that the number of subsequent papers in the three groups are *n*_*i*_, *n*_*j*_ and *n*_*k*_, respectively. Then the disruption score can be determined as

$$\begin{array}{c} D=\frac{n_{i}-n_{j}}{n_{i}+n_{j}+n_{k}} \end{array}$$ (1)

where *n*_*i*_ − *n*_*j*_ quantifies the extent to which the focal paper has eclipsed attention towards preceding papers, and *n*_*i*_ + *n*_*j*_ + *n*_*k*_ represents the total number of subsequent papers within the citation network.

According to the above definition, the disruption score spans from -1 to 1. A positive score indicates that the focal paper draws more attention from subsequent publications than its references. By definition, such a focal paper is more disruptive. If a focal paper is disruptive enough, then its disruption score *D* should be close to 1. Conversely, a negative score implies that the focal paper is more likely to be developmental. The focal paper exhibits an increasing degree of its developmental character as its disruption score approaches to -1. Overall, the disruption score not only allows us to quantify the disruption of each paper, but also to compare the disruption level among different publications.

We also note that the disruption score can be represented by an alternative formula given as

$$\begin{array}{c} D=\frac{1}{n}\sum_{i=1}^{n}-2f_{i}b_{i}+f_{i} \end{array}$$ (2)

where *n* denotes the total number of subsequent papers in the citation network, *i* represents the collection of subsequent works that cite the focal paper and/or the focal paper’s references, *f*_*i*_ = 1 if *i* only cites the focal paper and 0 otherwise, and *b*_*i*_ = 1 if *i* cites any predecessors of the focal paper and 0 otherwise.

These two expressions of the disruption score are mathematically equivalent. For the second expression, if a subsequent paper *i* cites only the focal paper, i.e., belonging to the *n*_*i*_ group, then −2*f*_*i*_*b*_*i*_ + *f*_*i*_ = 1 as −2*f*_*i*_*b*_*i*_ = 0. When a subsequent paper cites both the focal paper and its predecessors (within the *n*_*j*_ group), then the value of −2*f*_*i*_*b*_*i*_ + *f*_*i*_ will be -1. If a subsequent paper belongs to the *n*_*k*_ group, then −2*f*_*i*_*b*_*i*_ + *f*_*i*_ equals 0. By summing the −2*f*_*i*_*b*_*i*_ + *f*_*i*_ terms across all the subsequent papers in the citation network, the result equals to the difference between the number of *n*_*i*_ papers and *n*_*j*_ papers, which is the *n*_*i*_ − *n*_*j*_ term in the first formula.

Having introduced the mathematical expression of the disruption score, we now discuss several critical decisions regarding its application throughout this study. Most importantly, we calculate the disruption score of a paper using all subsequent publications, rather than adhering to the common practice of using those published within a 5-year time window [27, 29], i.e., the CD_5_ metric. We do this for three primary reasons. First, using an extended time window circumvents the issue of delayed recognition for disruptive research [39]. Second, incorporating the full publication record can yield a more accurate representation of a paper’s true disruption [63], as the most significant difference between citations and disruptions is that citations increase monotonically with time, whereas the disruption score varies depending on the citation behaviours of its subsequent works. Third, computing the disruption metric based on a small number of citations can result in an inflated score. To address this problem, we use the entire publication record to include more subsequent papers in the calculation of the disruption score.

Nevertheless, these decisions may subject the disruption metric to other biases, such as the overall decline in papers’ disruptions over time, the changing citation behaviours for papers published at different times, and the reduced comparability of the disruption metric across years. To mitigate these biases, we adopt two alternative versions of the disruption score and use them to validate all of our results throughout the study. The first version is the CD_5_ metric, which is computed only from papers published in the 5 years following the focal paper. The CD_5_ metric is frequently used in the literature [27, 29]. The second version is the standardized disruption score. We group papers by their respective publication years, and then standardize their disruption levels by incorporating the mean and standard deviation of that year’s distribution of disruption scores (i.e., transforming into z-scores). In this paper, our findings remain consistent in both the CD_5_ metric and standardized disruption scores, which further corroborates the validity of our results.

## Supporting information

S1 Fig The robustness check for Fig 1 under the CD_5_ metric. Left column: Correlation coefficients across cumulative percentiles of top disruptive papers in Computer Science (top) and Physics (bottom). Right column: The proportion of citations received by each percentile of Computer Science (top) and Physics (bottom) papers.
(TIF)

S2 Fig The robustness check for Fig 1 under the standardized disruption score. Left column: Correlation coefficients across cumulative percentiles of top disruptive papers in Computer Science (top) and Physics (bottom). Right column: The proportion of citations received by each percentile of Computer Science (top) and Physics (bottom) papers.
(TIF)

S3 Fig The robustness check for Fig 1 under the null model. We create the null model by reshuffling the 5-year accumulated citations received by each paper. Under the null model, the left column represents correlation coefficients across cumulative percentiles of top disruptive papers in Computer Science (top) and Physics (bottom), and the right column presents the proportion of citations received by each percentile of Computer Science (top) and Physics (bottom) papers.
(TIF)

S4 Fig The robustness check for Fig 3. Left column: Correlation coefficients across cumulative percentiles of most-cited papers in Computer Science (top) and Physics (bottom) under the CD_5_ metric. Center column: Correlation trajectories for most-cited papers in Computer Science (top) and Physics (bottom) under the standardized disruption score. Right column: Correlation trajectories for most-cited papers in Computer Science (top) and Physics (bottom) under the null model.
(TIF)

S5 Fig Proportion of citations received by researchers with more than 100 publications. Mean values of proportion of citations for each percentile of disruptive papers in the publication profile of Computer Scientists (top) and Physicists (bottom). The plots are constructed based on researchers who have more than 100 publications. We can see that these figures are similar to the curves in (b) and (d) of Fig 1.
(TIF)

S6 Fig The robustness check for Fig 4 under the CD_5_ metric. Left column: mean values of correlation coefficients across cumulative percentiles of top disruptive papers in the publication sequences of Computer Scientists (top) and Physicists (bottom). Right column: mean values of proportion of citations received by each percentile of papers in the publication profile of Computer Scientists (top) and Physicists (bottom).
(TIF)

S7 Fig The robustness check for Fig 4 under the standardized disruption score. Left column: mean values of correlation coefficients across cumulative percentiles of top disruptive papers in the publication sequences of Computer Scientists (top) and Physicists (bottom). Right column: mean values of proportion of citations received by each percentile of papers in the publication profile of Computer Scientists (top) and Physicists (bottom).
(TIF)

S8 Fig The robustness check for Fig 4 under the null model. Left column: mean values of correlation coefficients across cumulative percentiles of top disruptive papers in the publication sequences of Computer Scientists (top) and Physicists (bottom). Right column: mean values of proportion of citations received by each percentile of papers in the publication profile of Computer Scientists (top) and Physicists (bottom). It is apparent that our results in Fig 4 cannot be explained by the null models.
(TIF)

S9 Fig The robustness check for Fig 5. Left column: mean values of correlation coefficients across cumulative percentiles of most-cited papers papers in the publication sequences of Computer Scientists (top) and Physicists (bottom), as measured by the CD_5_ metric. Center column: mean values of correlation coefficients for most-cited papers in the publication sequences of Computer Scientists (top) and Physicists (bottom), as measured by the standardized disruption score. Right column: mean values of correlation coefficients for most-cited papers in the publication sequences of Computer Scientists (top) and Physicists (bottom) under the null model.
(TIF)
