# Supplementary figures and images for: Breaking down the relationship between disruption scores and citation counts
Source: PLoS One. 2024 Dec 19;19(12):e0313268. doi: 10.1371/journal.pone.0313268 (PMC11658639; doi:10.1371/journal.pone.0313268)

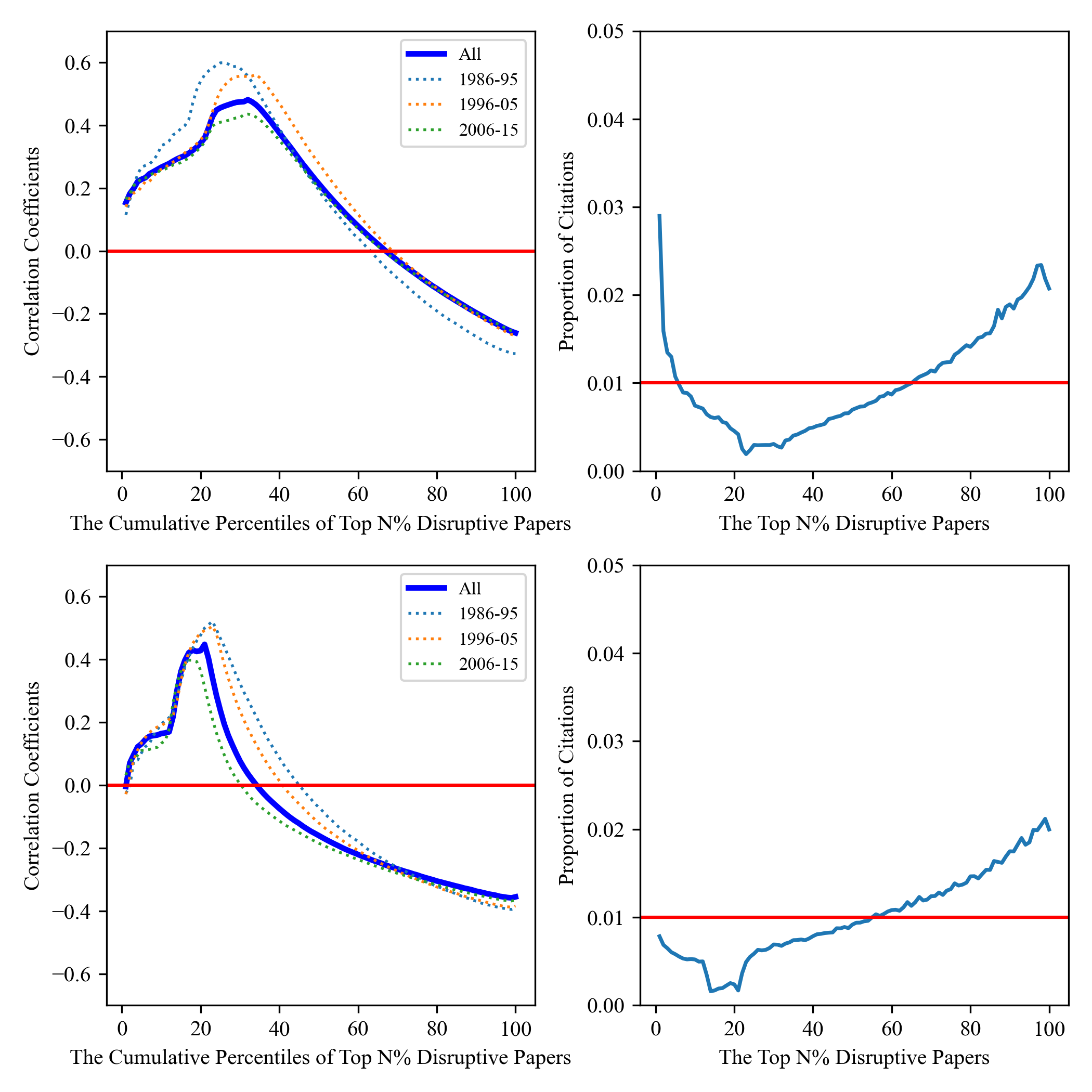

Supplement: S1 Fig — Left column: Correlation coefficients across cumulative percentiles of top disruptive papers in Computer Science (top) and Physics (bottom). Right column: The proportion of citations received by each percentile of Computer Science (top) and Physics (bottom) papers. (TIF) [file pone.0313268.s001.tif]

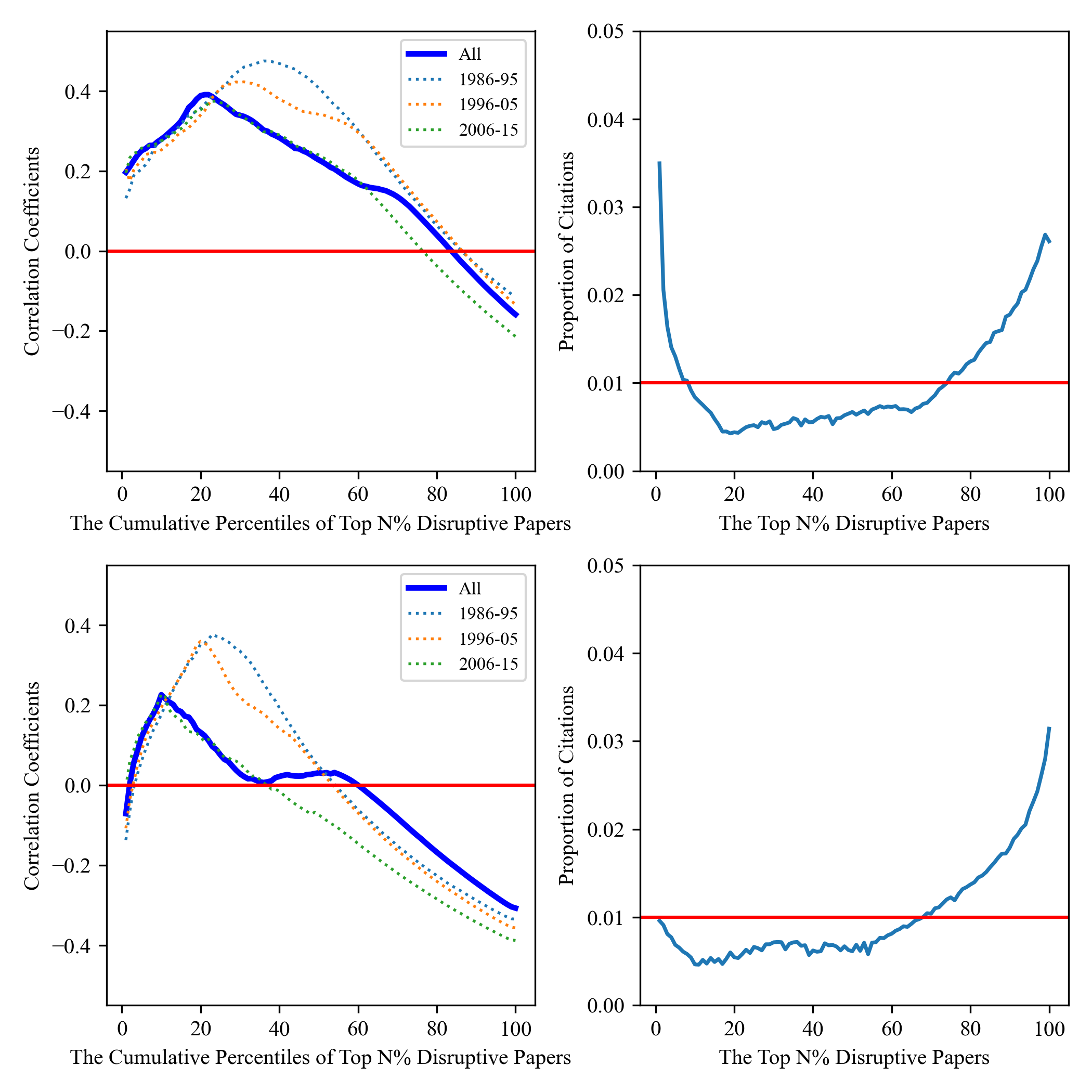

Supplement: S2 Fig — Left column: Correlation coefficients across cumulative percentiles of top disruptive papers in Computer Science (top) and Physics (bottom). Right column: The proportion of citations received by each percentile of Computer Science (top) and Physics (bottom) papers. (TIF) [file pone.0313268.s002.tif]

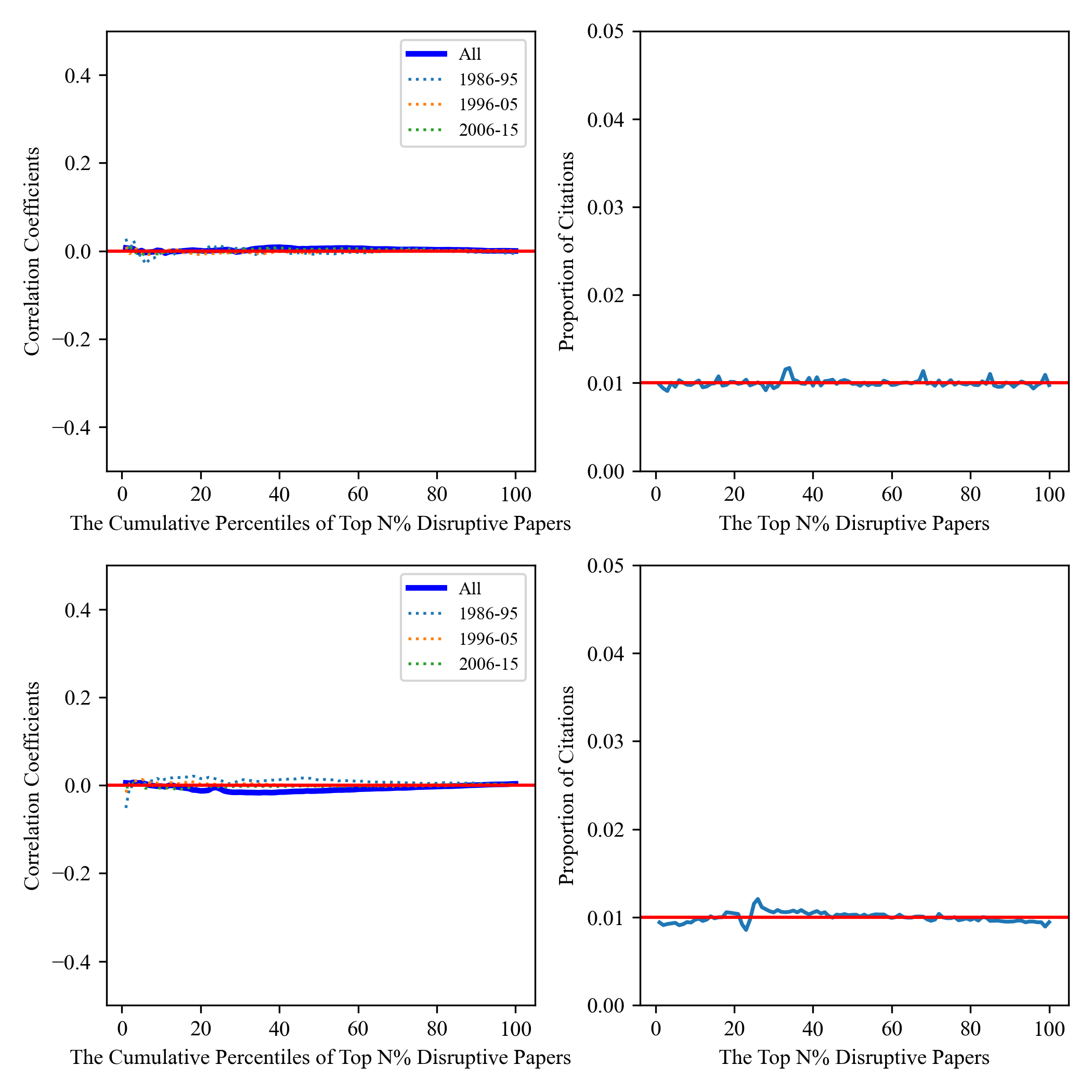

Supplement: S3 Fig — We create the null model by reshuffling the 5-year accumulated citations received by each paper. Under the null model, the left column represents correlation coefficients across cumulative percentiles of top disruptive papers in Computer Science (top) and Physics (bottom), and the right column presents the proportion of citations received by each percentile of Computer Science (top) and Physics (bottom) papers. (TIF) [file pone.0313268.s003.tif]

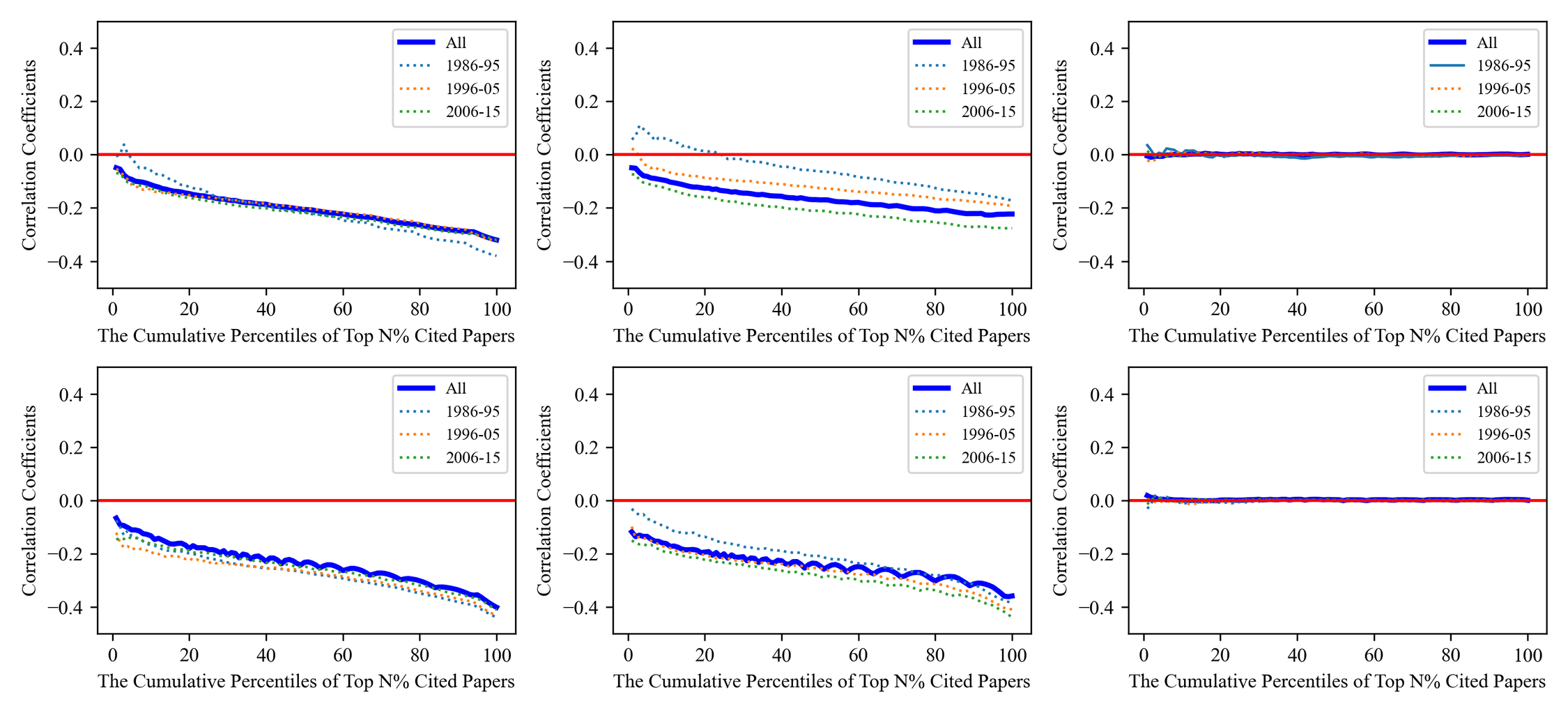

Supplement: S4 Fig — Left column: Correlation coefficients across cumulative percentiles of most-cited papers in Computer Science (top) and Physics (bottom) under the CD5 metric. Center column: Correlation trajectories for most-cited papers in Computer Science (top) and Physics (bottom) under the standardized disruption score. Right column: Correlation trajectories for most-cited papers in Computer Science (top) and Physics (bottom) under the null model. (TIF) [file pone.0313268.s004.tif]

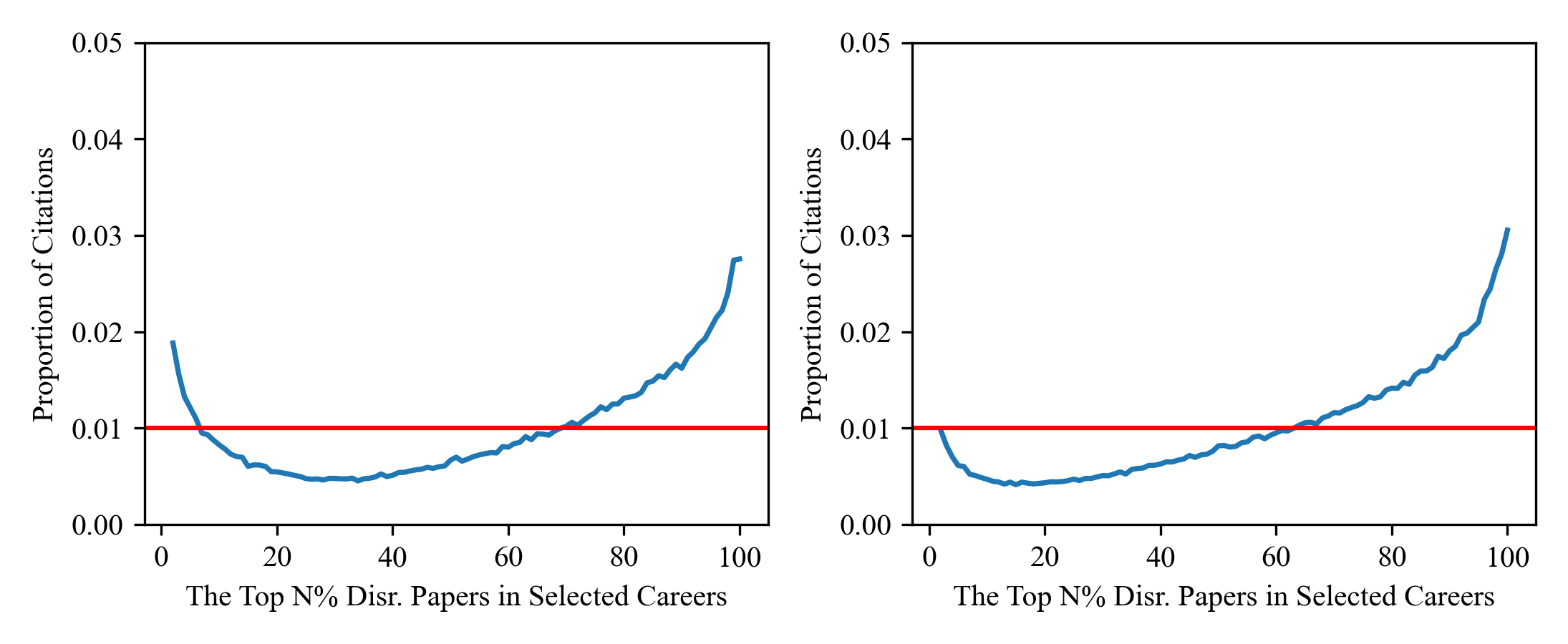

Supplement: S5 Fig — Mean values of proportion of citations for each percentile of disruptive papers in the publication profile of Computer Scientists (top) and Physicists (bottom). The plots are constructed based on researchers who have more than 100 publications. We can see that these figures are similar to the curves in (b) and (d) of Fig 1. (TIF) [file pone.0313268.s005.tif]

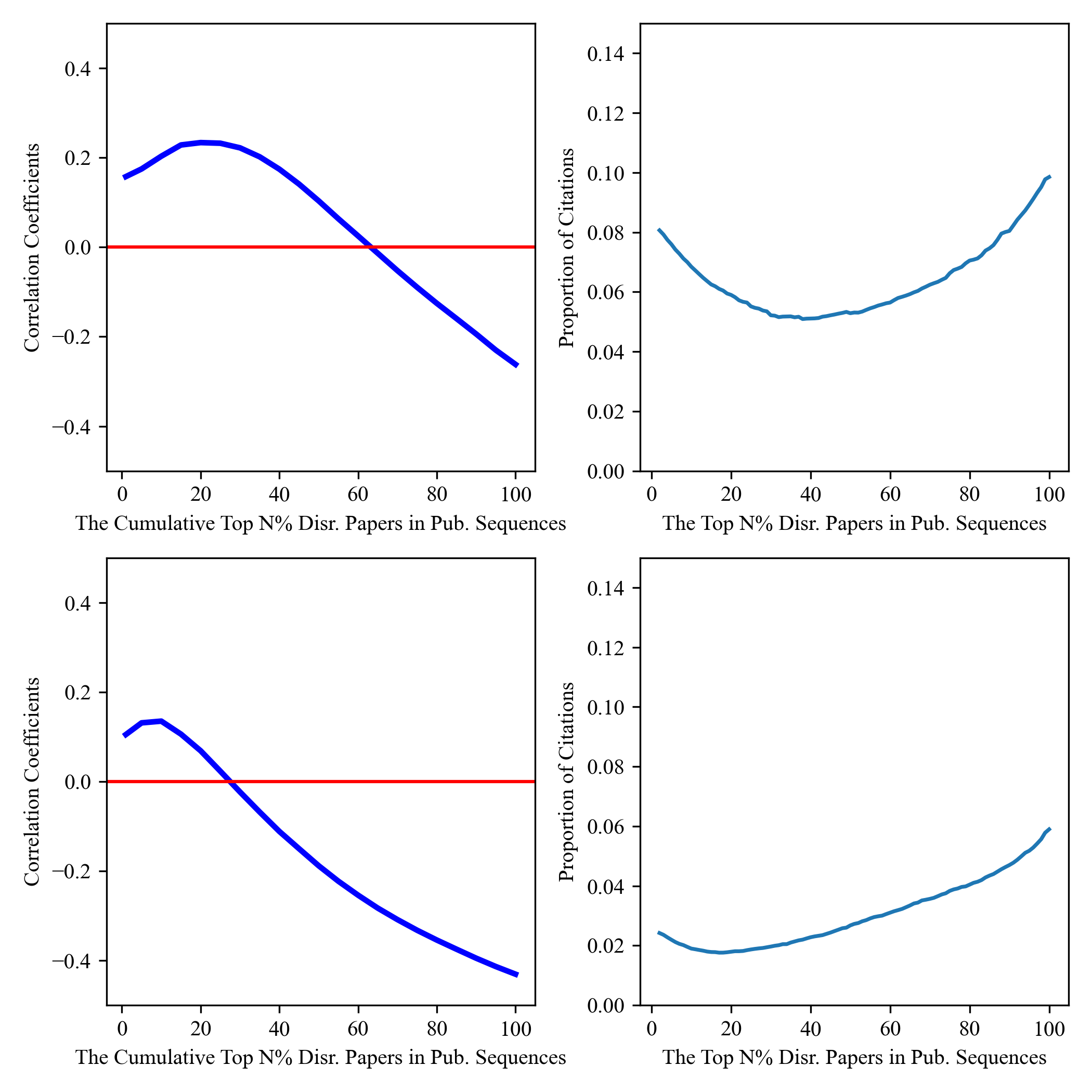

Supplement: S6 Fig — Left column: mean values of correlation coefficients across cumulative percentiles of top disruptive papers in the publication sequences of Computer Scientists (top) and Physicists (bottom). Right column: mean values of proportion of citations received by each percentile of papers in the publication profile of Computer Scientists (top) and Physicists (bottom). (TIF) [file pone.0313268.s006.tif]

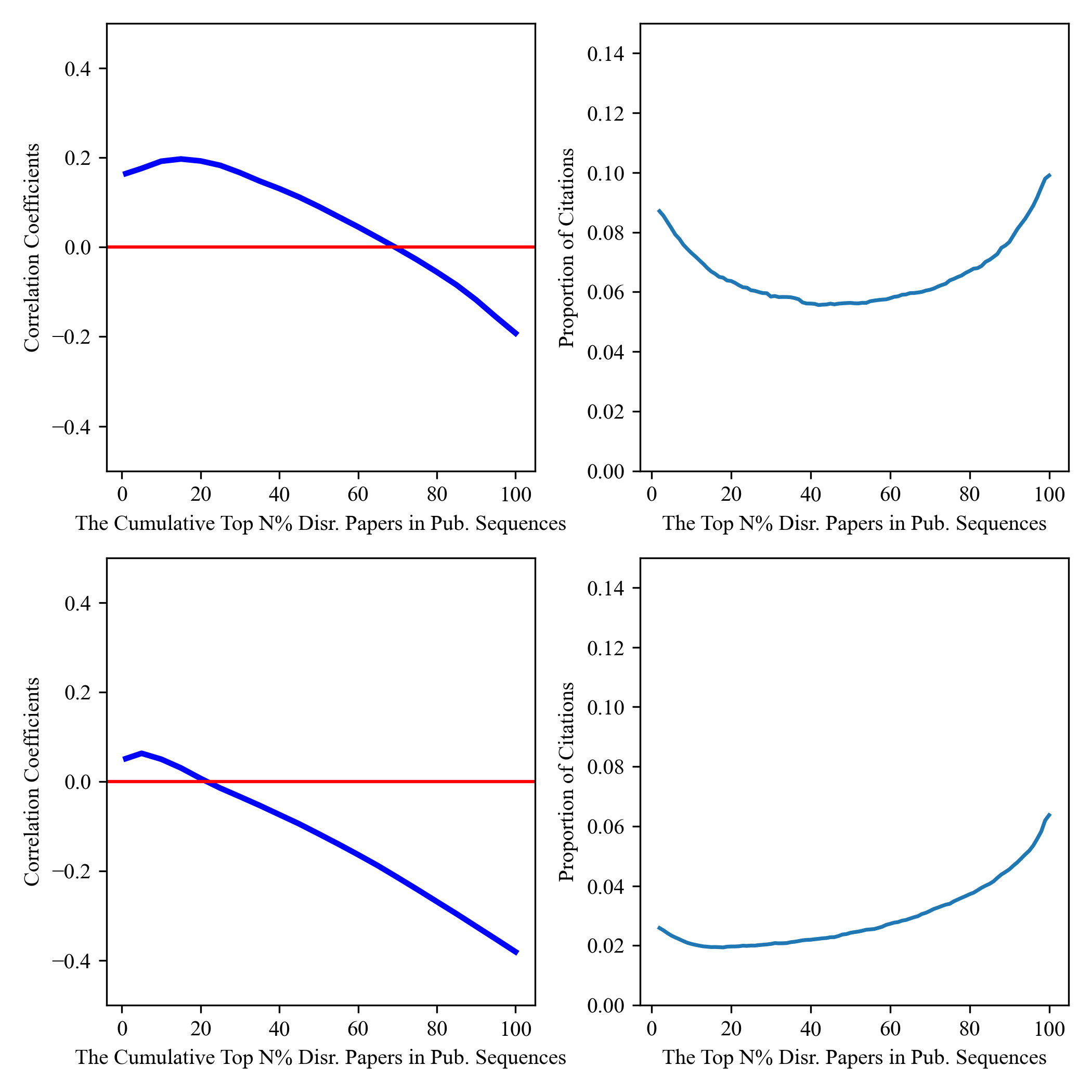

Supplement: S7 Fig — Left column: mean values of correlation coefficients across cumulative percentiles of top disruptive papers in the publication sequences of Computer Scientists (top) and Physicists (bottom). Right column: mean values of proportion of citations received by each percentile of papers in the publication profile of Computer Scientists (top) and Physicists (bottom). (TIF) [file pone.0313268.s007.tif]

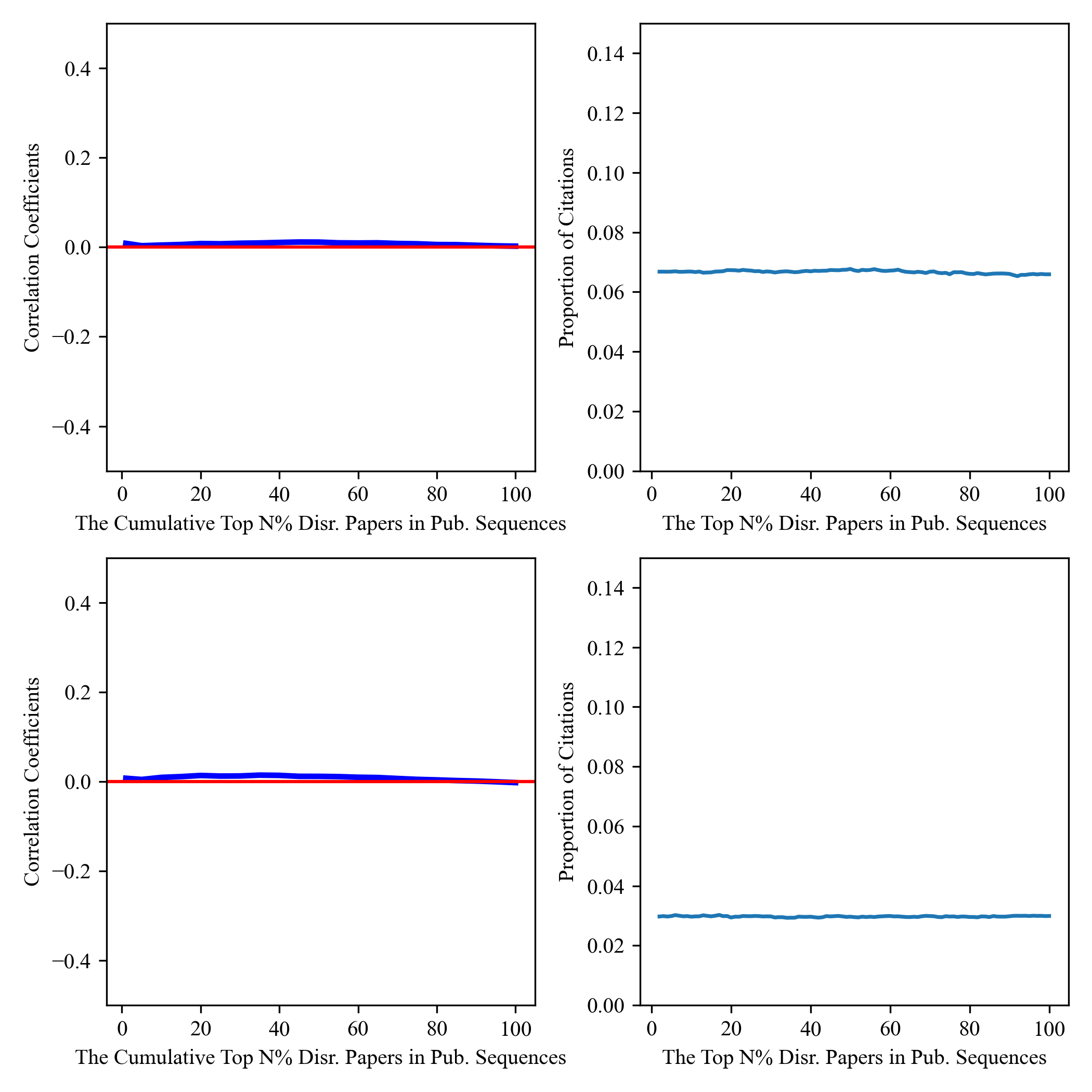

Supplement: S8 Fig — Left column: mean values of correlation coefficients across cumulative percentiles of top disruptive papers in the publication sequences of Computer Scientists (top) and Physicists (bottom). Right column: mean values of proportion of citations received by each percentile of papers in the publication profile of Computer Scientists (top) and Physicists (bottom). It is apparent that our results in Fig 4 cannot be explained by the null models. (TIF) [file pone.0313268.s008.tif]

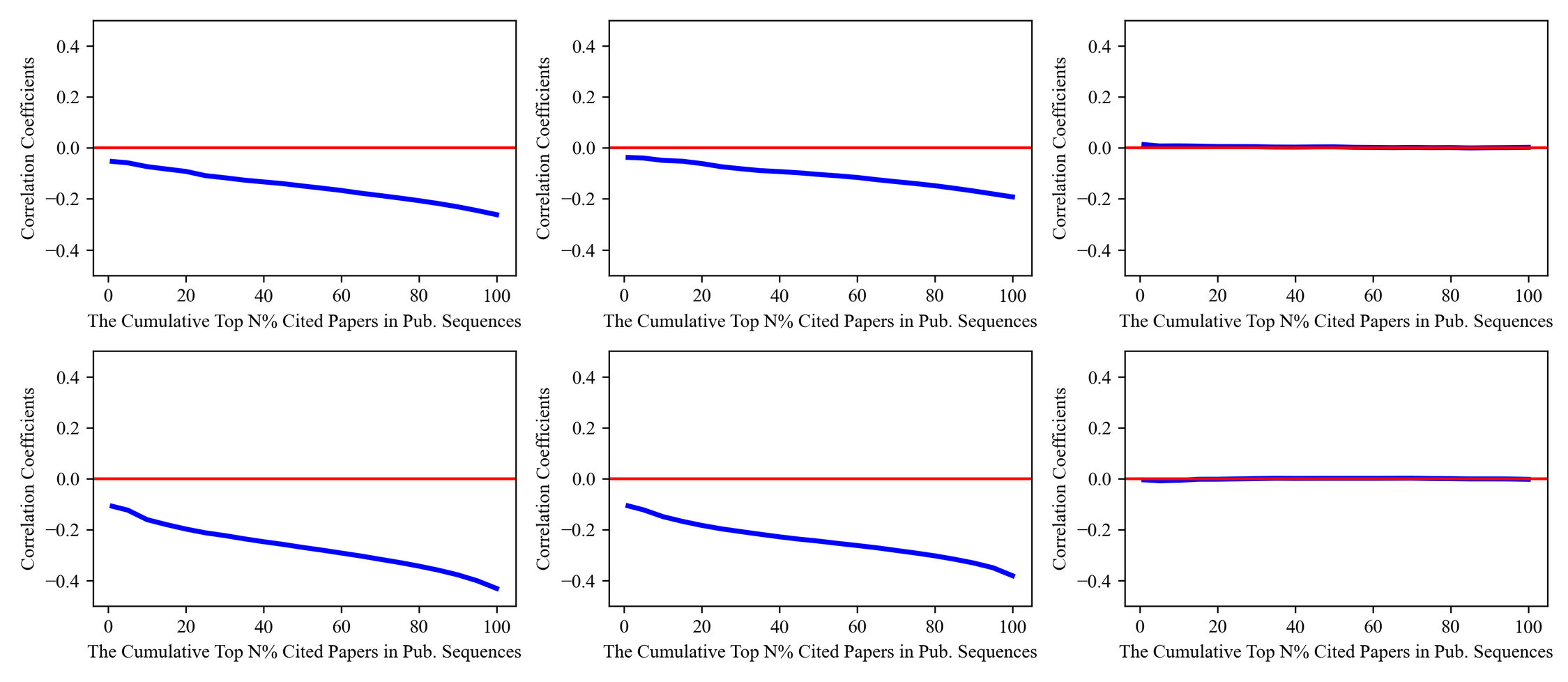

Supplement: S9 Fig — Left column: mean values of correlation coefficients across cumulative percentiles of most-cited papers papers in the publication sequences of Computer Scientists (top) and Physicists (bottom), as measured by the CD5 metric. Center column: mean values of correlation coefficients for most-cited papers in the publication sequences of Computer Scientists (top) and Physicists (bottom), as measured by the standardized disruption score. Right column: mean values of correlation coefficients for most-cited papers in the publication sequences of Computer Scientists (top) and Physicists (bottom) under the null model. (TIF) [file pone.0313268.s009.tif]
